# Genetic diversity and phylogenetic characteristics of viruses in lily plants in Beijing

**DOI:** 10.3389/fmicb.2023.1127235

**Published:** 2023-04-17

**Authors:** Ling Chen, Cheng Guo, Chenge Yan, Rui Sun, Yongqiang Li

**Affiliations:** ^1^School of Grassland Science, Beijing Forestry University, Beijing, China; ^2^College of Biological Science and Resources Environment, Beijing University of Agriculture, Beijing, China

**Keywords:** lily virome, lily endornavirus, lily polerovirus, diversity, recombination

## Abstract

Lily (*Lilium*) is an important bulbous perennial herb that is frequently infected by one or more viruses. To investigate the diversity of lily viruses, lilies with virus-like symptoms in Beijing were collected to perform small RNA deep sequencing. Then, the 12 complete and six nearly full-length viral genomes, including six known viruses and two novel viruses were determined. Based on sequence and phylogenetic analyses, two novel viruses were considered to be members of the genera *Alphaendornavirus* (*Endornaviridae*) and *Polerovirus* (*Solemoviridae*). These two novel viruses were provisionally named lily-associated alphaendornavirus 1 (LaEV-1) and lily-associated polerovirus 1 (LaPV-1). Based on sequence, phylogenetic and recombination analyses, strawberry latent ringspot virus (SLRSV) in the genus *Stralarivirus* (*Secoviridae*) was identified for the first time in China, and shown to exhibit the highest nucleotide (nt) diversity among the available full-length SLRSV genome sequences, with the highest identities of 79.5% for RNA1 and 80.9% for RNA2. Interestingly, the protease cofactor region in RNA1 was 752 aa in length, whereas those of the other 27 characterized isolates ranged from 700–719 aa in length. The genome sequences of lily virus A (*Potyvirus*), lily virus X (*Potexvirus*), and plantago asiatica mosaic virus (*Potexvirus*) exhibited varying degrees of sequence diversity at the nucleotide level compared with their corresponding characterized isolates. In addition, plantago asiatica mosaic virus (PlAMV) tended to cluster on a host species-basis. One identified lily mottle virus (*Potyvirus*) isolate was detected as a recombinant, and which clustered in a different group with four other isolates. Seven identified lily symptomless virus (*Carlavirus*) isolates, including one recombinant, were clustered into three clades. Our results revealed the genetic diversity of lily-infecting viruses, and sequence insertion, host species and recombination are factors that likely contribute to this diversity. Collectively, our results provide useful information regarding the control of viral disease in lily.

## 1. Introduction

Lily (*Lilium*) is an important bulbous perennial herb from the *Liliaceae* family of the monocotyledonous subclass that is cultivated as an ornamental plant used for cut flower production, as potted plants, and for park, garden, and landscape decoration throughout the world. Lily bulbs are also used as food and medicine in several Asian cultures (Grassotti and Gimelli, 2011; Qu et al., 2014). There are more than 4,500 cultivated lily varieties recorded to date (Sharma et al., 2005).

Lilies are usually vegetatively propagated using bulbs and scaling, which involves detaching scales from the bulb and planting individual scales to generate new bulbs (Simmonds and Cumming, 1976; Jo and Cho, 2018). This type of vegetative propagation is an efficient way for virus transmission (Sharma et al., 2005; Zhang et al., 2017). To date, more than 15 lily-infecting viruses have been reported, including lily symptomless virus (LSV), lily mottle virus (LMoV), cucumber mosaic virus (CMV), plantago asiatica mosaic virus (PlAMV), tulip virus X (TVX), tobacco rattle virus (TRV) (Jo and Cho, 2018), lily yellow mosaic virus (LYMV) (Li et al., 2017), strawberry latent ringspot virus (SLRSV) (Sharma et al., 2005), lily virus X (Nijo et al., 2018), arabis mosaic virus (ArMV) (Zheng et al., 2003), prunus necrotic ringspot virus (PNRSV) (Han and Liu, 2007), lily virus A (Wylie et al., 2012), shallot yellow stripe virus (SYSV) (Xu et al., 2022), tomato ringspot virus (ToRSV) (Bellardi et al., 2002), milk retch dwarf virus (MDV) (Choi et al., 2019), and narcissus mosaic virus (NaMV) (Bertaccini, 1988). Among these viruses, only MDV is a single-stranded DNA virus, and the other 15 viruses are positive-sense RNA viruses. LSV, LMoV, CMV, and PlAMV are widely distributed worldwide, and have been reported in the Netherlands (Asjes, 2000), the United States (Hammond et al., 2015), Italy (Parrella et al., 2015), Japan (Niimi et al., 2003), Korea (Sharma et al., 2005), and China (Zhang et al., 2018) in recent decades. Other lily-infecting viruses have been reported to occur only in some regions. In Korea, the incidence of LSV has reached an infection rate of 45.0% (Kim et al., 2015). In China, LSV, CMV, and LMoV are also widely distributed in major lily-production regions including Gansu, Ningxia, Qinghai, Jiangsu, and Yunnan provinces. The incidence of LSV, CMV, and LMoV in Lanzhou lily (*Lilium davidii* var. *unicolor*), a popular edible vegetable bulb, has reached infection rates of 98.6, 34.3, and 32.9%, respectively (Zhang et al., 2018). Infection with these viruses can decrease Lanzhou lily production by 50% (Zhang et al., 2015). In addition, it has been reported that PlAMV infection can render 80% of glasshouse-grown lily plants unsuitable for harvesting (Tanaka et al., 2019). The above viruses detected in lily are associated with the symptoms of vein clearing, leaf mottle, mosaic, chlorosis, necrotic spots, streaking, and/or leaf deformation, which significantly reduce the commercial value of lily as an ornamental plant (Asjes, 2000; Kim et al., 2012). In many cases, these symptoms of lily are usually associated with infection by more than one virus.

High-throughput sequencing (HTS) has been shown to be a fast and sensitive technique widely used in virus discovery (Wu et al., 2015; Villamor et al., 2019; Lee et al., 2022). Using this technique, several novel viruses have been identified as the causal agents of some important viral diseases (Ma et al., 2015; Cheng et al., 2022). In addition, this technique has been used to study plant viromes, which could provide significant information on viral populations and viral quasispecies (Jo et al., 2017; Jo and Cho, 2018; Liu et al., 2021). The HTS of small RNAs, also known as small RNA sequencing (sRNA-seq), can detect all types of viruses (DNA and RNA) rapidly, and the obtained viral genome sequences are easily to be assembled, except for viruses that produce small amounts of sRNAs (Wu et al., 2010).

In this study, sRNA-seq was performed to study the viromes associated with the observed virus-like disease in lily plants in Beijing. A total of 12 complete and six nearly full-length viral genomes, including two novel and six known lily-infecting viruses, were determined and characterized. In addition, the recombination and phylogeny of these viruses were analyzed. The genetic diversity and phylogenetic characteristic data generated in this study will contribute to understanding the taxonomy, variation and evolution of lily-infecting viruses and the design of better diagnostic assays for use in clean plant programs for lily propagation. Consequently, these findings will enhance our capacity to control viral disease affecting lily cultivation.

## 2. Materials and methods

### 2.1. Plant materials

Leaf samples from 24 lily plants, exhibiting the symptoms of mosaic, yellowing, leafroll, and stunted growth, were collected in June 2019 from Beijing Botanical Garden and Beijing Yanqing Expo Park, quickly frozen in liquid nitrogen, and stored at −80°C before use. Total RNA was extracted from leaf samples of each symptomatic plant using TRIzol reagent (Invitrogen, Carlsbad, CA, USA) as recommended by the manufacturer. Then, the quality and quantity of the RNA were assessed using a 2100 Bioanalyzer (Agilent Technologies, Waldbroon, Germany) and a NanoDrop ND-1000 (Nanodrop Technologies, Wilmington, DE, USA), respectively. The high-quality RNA was subsequently used for sRNA-seq.

### 2.2. Small RNA sequencing

Four individual RNA samples were equally pooled into a sequenced sample according to the location and symptom of the sampled plants, and eventually, six small RNA libraries were constructed (Table 1). These small RNA libraries were constructed using a TruSeq Small RNA Sample Prep Kit (Illumina, San Diego, CA, USA). Typically, small RNAs of 18–28 nucleotides (nt) in length were isolated from the total RNA samples using 15% polyacrylamide gels (Invitrogen, Carlsbad, CA, USA), and subjected to 5′ and 3′ adaptor ligation. Subsequently, the final ligation products were purified and reverse-transcribed, followed by PCR enrichment. The quality of the resulting libraries was assessed using a 2100 Bioanalyzer, and they were sequenced on the Illumina HiSeq4000 platform. The raw sequencing data has been deposited in the Sequence Read Archive (SRA) with accession number PRJNA935951. Then, the raw data were processed to trim adapters and remove sequences shorter than 18 nt or longer than 26 nt, or sequences with low-quality and sequences containing polyA tags using the Cutadapt v.1.17 tool (Martin, 2011). The resulting clean reads were subjected to BLAST searches against the GenBank Virus RefSeq database, and the sRNAs mapped to the viruses were assembled using PFOR (Wu et al., 2012) and Velvet (Wu et al., 2010) software to obtain the contigs. The obtained contigs were subjected to BLASTn and BLASTx searches of the GenBank database to identify candidate viruses.

**TABLE 1:** The information of sRNA-seq and candidate viruses in each sequenced lily plant.

Sampling locations	Library name	Clean reads	Number of contigs	Viral species (number of contigs)	Plant symptoms	LaEV-1	LaPV-1	SLRSV	LVA	LVX	PlAMV	LMoV	LSV	CMV
Beijing Botanical Garden	Lily sRNA 11A	6,200,030	9,047	LMoV (241), LSV (122), CMV (225), secovirus (77), Caulimoviridae (22)	Mosaic							**+**	**+**	**+**
					Mosaic, leafroll			**+**				**+**	**−**	**+**
					Leafroll							**+**	**+**	**+**
					Mosaic, leafroll							**+**	**+**	**+**
	Lily sRNA 18A	5,281,949	7,448	LMoV (196), LSV (110), CMV (217), PlAMV (27), Caulimoviridae (23)	Mosaic, leafroll						**+**	**+**	**−**	**−**
					Yellowing, leafroll							**−**	**+**	**+**
					Mosaic, leafroll						**+**	**−**	**+**	**+**
					Mosaic, leafroll							**+**	**−**	**+**
Beijing Yanqing Expo Park	Lily sRNA 15A	6,939,853	10,890	LMoV (184), LVA (36), CMV (191), PlAMV(3), Caulimoviridae (22)	Yellowing, leafroll							**+**	**−**	**+**
					Mosaic, leafroll						**+**	**+**	**+**	**−**
					Mosaic, leafroll							**+**	**−**	**+**
					Leafroll				**+**			**−**	**−**	**+**
	Lily sRNA 17A	7,812,994	10,836	LMoV (273), CMV (229), LSV (6), endornavirus (7), PlAMV (5), Caulimoviridae (28)	Yellowing, mosaic, leafroll							**+**	**+**	**−**
					Yellowing, mosaic, leafroll							**+**	**−**	**+**
					Yellowing, mosaic, leafroll, stunt	**+**					**+**	**+**	**−**	**+**
					Yellowing, mosaic, leafroll, stunt							**+**	**+**	**+**
	Lily sRNA 7A	10,570,270	16,943	LMoV (312), LSV (82), CMV (226), LVX (64), PlAMV (68), Caulimoviridae (33)	Yellowing, leafroll						**+**	**−**	**−**	**+**
					Yellowing, leafroll					**+**		**+**	**+**	**−**
					Yellowing, mosaic						**+**	**+**	**−**	**+**
					Mosaic							**+**	**+**	**+**
	Lily sRNA 8A	6,975,756	11,148	LMoV (258), LSV (57), CMV (170), polerovirus (25), Caulimoviridae (14)	Yellowing, mosaic, leafroll		**+**					**+**	**−**	**+**
					Yellowing, mosaic, stunt							**+**	**+**	**−**
					Yellowing, mosaic							**+**	**−**	**+**
					Yellowing, mosaic							**+**	**−**	**+**
Total					24	1	1	1	1	1	6	20	11	19
Detection rate (%)						4.2	4.2	4.2	4.2	4.2	25.0	83.3	45.8	79.2

LaEV-1, lily-associated endornavirus 1; LaPV-1, lily-associated polerovirus 1; SLRSV, strawberry latent ringspot virus; LVA, lily virus A; LVX, lily virus X; PlAMV, plantago asiatica mosaic virus; LMoV, lily mottle virus; LSV, lily symptomless virus; CMV, cucumber mosaic virus.

### 2.3. Genome sequence reconstruction and virus detection

The complete genome sequences of the candidate viruses detected from small RNA sequencing data were determined by overlapping RT-PCR using primer pairs designed from contig sequences (Supplementary Table 1). The determination of the candidate viruses in each sequenced plant was performed by RT-PCR using primer pairs designed from the reconstructed genome sequences (Supplementary Table 1). More specifically, the first-strand cDNA was synthesized using M-MLV reverse transcriptase according to the instructions of the manufacturer (Promega, Madison, WI, USA). The reverse transcription reaction mixture (20 μL final volume) included 5 μL of total RNA (2.5 μg), 4 μL of dNTP mixture (2.5 mM), 1 μL (200 U) of M-MLV reverse transcriptase, 4 μL of M-MLV 5 × reaction buffer, 1 μL of random primers (10 μM), 1 μL (40 U) of recombinant RNasin ribonuclease inhibitor, and 4 μL of sterile, RNase-free water. The mixture was incubated at 37°C for 60 min. After the reverse transcription reaction, 1 μL cDNA product was added to 24 μL of the PCR mixture which consisted of 12.5 μL Premix *Taq* (*LA Taq*, Takara Bio Inc., Dalian, China), 0.5 μL of forward primer (10 μM), 0.5 μL of reverse primer (10 μM), and 10.5 μL of sterile distilled water. The amplified sequences were cloned into the pMD18-T vector (TaKaRa, Dalian, China). Sequences of the 5′ and 3′-untranslated regions (UTRs) were amplified using a SMARTer rapid amplification of cDNA ends (RACE) kit (TaKaRa, Dalian, China). At least five independent clones of each fragment used for genome sequence reconstruction were sequenced by Sangon Biotech (Shanghai) Co., Ltd.

The sequences were then assembled using DNAMAN 6.0 software. The open reading frames (ORFs) of the candidate lily-infecting viruses were identified and analyzed according to the translational strategy of viruses in their family and by using “ORF Finder” at NCBI.^1^ Conserved and functional protein domains were identified using the Conserved Domain Database at NCBI.^2^

### 2.4. Sequence, recombination, and phylogenetic analyses

Sequence similarity searches were performed using the online NCBI BLAST tool. The detection of recombination events was performed with various recombination detection methods implemented in the software RDP 4 (Martin et al., 2015), including the programs RDP, GENECONV, BOOTSCAN, MAXCHI, CHIMAERA, SISCAN, and 3SEQ using an alignment of all available full-length virus sequences constructed with ClustalX2. All analyses were performed under the default configuration. Potential recombination events detected were considered to be statistically significant if detected by four or more different programs, two or more types of methods (BootScan, RDP, and SiScan were based on phylogenetic methods; GENECONV, MaxChi, Chimaera, and 3Seq were based on substitution methods), and with a *P*-value < 1.0 × 10^–6^ (Ohshima et al., 2007).

The phylogenetic tree was constructed by the maximum-likelihood (ML) and neighbor-joining (NJ) methods (1,000 bootstrapping replicates) with the program MEGA 6 (Tamura et al., 2013). The best-fit protein substitution model for the RNA dependent RNA polymerase (RdRp) of lily-associated alphaendornavirus 1 (LaEV-1) was LG + G + I (Gama Distributed with Invariable Sites), and the best models for the RdRp and coat protein (CP) of lily-associated polerovirus 1 (LaPV-1) were LG + G and WAG + G, respectively, determined using the function in MEGA6. The best-fit nucleotide substitution model for the full-length genome sequences of PlAMV was GTR (General Time Reversible) + G + I determined using the function in MEGA6. In addition, the best-fit protein substitution model for all other detected proteins was determined to be JTT (Jones–Taylor–Thornton) + G using the function in MEGA6.

## 3. Results

### 3.1. Confirmation of the presence of candidate viruses

Leaf samples of 24 lily plants were used to construct six sRNA sequencing libraries according to their symptoms and locations. BLAST analyses using the contigs generated from these libraries revealed the possible existence of at least eight lily-infecting viral species, including LVA, PlAMV, LVX, LMoV, LSV, and three unknown viruses, including an endornavirus, a polerovirus, and a secovirus. The possible existence of viruses in the *Caulimoviridae* family was also indicated (Table 1). To confirm the full-length genome sequences of the viruses detected from the small RNA sequencing data, overlapping RT-PCR and RACE were performed using the primer pairs listed in Supplementary Table 1. As a result, 12 complete and six nearly complete genome sequences for six known and two novel lily-infecting viruses were determined and deposited in GenBank (Table 2). The two novel viruses identified were provisionally designated LaEV-1 and LaPV-1. The highest nucleotide identities of the six known viruses were 74.74–98.73% compared with their corresponding characterized isolates (Table 2), indicating the presence of molecular diversity in the populations of these lily-infecting viruses.

**TABLE 2:** The information of assembled genomes of lily-infecting viruses.

Virus	Library name	Plant symptom	Genus	Family	Isolate	Genome	Size (nt)	Accession no.	Top hit ID	%ID	%Cov
LaEV-1	Lily sRNA 17A	Yellowing, mosaic, leafroll, stunt	*Alphaendornavirus*	*Endornaviridae*	BJ	Complete	16,483	MZ614632			
LaPV-1	Lily sRNA 8A	Yellowing, mosaic, leafroll	*Polerovirus*	*Solemoviridae*	BJ	Partial	4,201	OQ024902			
SLRSV RNA1	Lily sRNA 11A	Mosaic, leafroll	*Stralarivirus*	*Secoviridae*	BJ	Complete	7,274	OM201233	MH237605	79.51	93
SLRSV RNA2					BJ	Complete	3,613	OM311160	MG062674	80.93	97
PlAMV	Lily sRNA 18A	Mosaic, leafroll	*Potexvirus*	*Alphaflexiviridae*	BJ	Complete	6,100	OM201231	KX245539	99.57	99
LVA	Lily sRNA 15A	Leafroll	*Potyvirus*	*Potyviridae*	BJ	Partial	7,900	OM201232	JN127335	98.28	100
LVX	Lily sRNA 7A	Yellowing, leafroll	*Potexvirus*	*Alphaflexiviridae*	BJ	Complete	5,822	OM311166	LC335818	98.85	100
LSV	Lily sRNA 8A	Yellowing, mosaic, stunt	*Carlavirus*	*Quinvirinae*	BJ-1	Partial	8,328	OM311154	AJ564638	98.41	100
LSV	Lily sRNA 11A	Mosaic			BJ-2	Complete	8,394	OM311155	AJ516059	98.23	100
LSV	Lily sRNA 18A	Yellowing, leafroll			BJ-3	Complete	8,393	OM311167	AJ516059	98.61	100
LSV	Lily sRNA 15A	Mosaic, leafroll			BJ-4	Partial	8,328	OM311156	AJ564638	98.29	100
LSV	Lily sRNA 17A	Yellowing, mosaic, leafroll, stunt			BJ-5	Partial	8,328	OM311157	GU440579	98.61	100
LSV	Lily sRNA 7A	Yellowing, leafroll			BJ-6	Complete	8,394	OM311158	AJ516059	98.73	100
LSV	Lily sRNA 11A	Leafroll			BJ-7	Partial	8,328	OM311159	HM222522	97.86	100
LMoV	Lily sRNA 18A	Mosaic, leafroll	*Potyvirus*	*Potyviridae*	BJ-1	Complete	9,629	OM311161	AB570195	99.50	99
LMoV	Lily sRNA 7A	Yellowing, leafroll			BJ-2	Complete	9,645	OM311162	AB570195	99.08	99
LMoV	Lily sRNA 8A	Yellowing, mosaic, leafroll			BJ-3	Complete	9,629	OM311163	AB570195	98.02	99
LMoV	Lily sRNA 11A	Mosaic, leafroll			BJ-4	Complete	9,641	OM311164	MT795719	97.73	99
LMoV	Lily sRNA 17A	Yellowing, mosaic, leafroll, stunt			BJ-5	Complete	9,626	OM311165	AB570195	99.23	99

ID, identity for GenBank accession number; Cov, coverage. Blue shading, proportional to the value, highlights divergent isolates.

To explore the distribution of the eight candidate viruses in 24 sampled lily plants from two sampled locations, RT-PCR was performed with a specific primer pair for each virus (Supplementary Table 1). The results showed that LMoV (83.3%) and CMV (79.2%) were the most prevalent viruses, followed by LSV (45.8%) and PlAMV (25.0%). LaEV-1, LaPV-1, SLRSV, LVA, and LVX were detected in only one plant each, in co-infections with other lily-infecting viruses in all cases (Table 1). Unfortunately, co-infection was identified in 24 detected plants with different typical symptoms, and in one sample, four viruses were detected together, resulting in difficulty in associating the observed symptoms with a lily-infecting virus (Table 1). These results also suggested that symptoms of lily plants were very complicated, which could be involved in the high prevalence of viruses and the existence of severe mixed infections.

### 3.2. Characterization of one novel alphaendornavirus from lily

**FIGURE 1:**
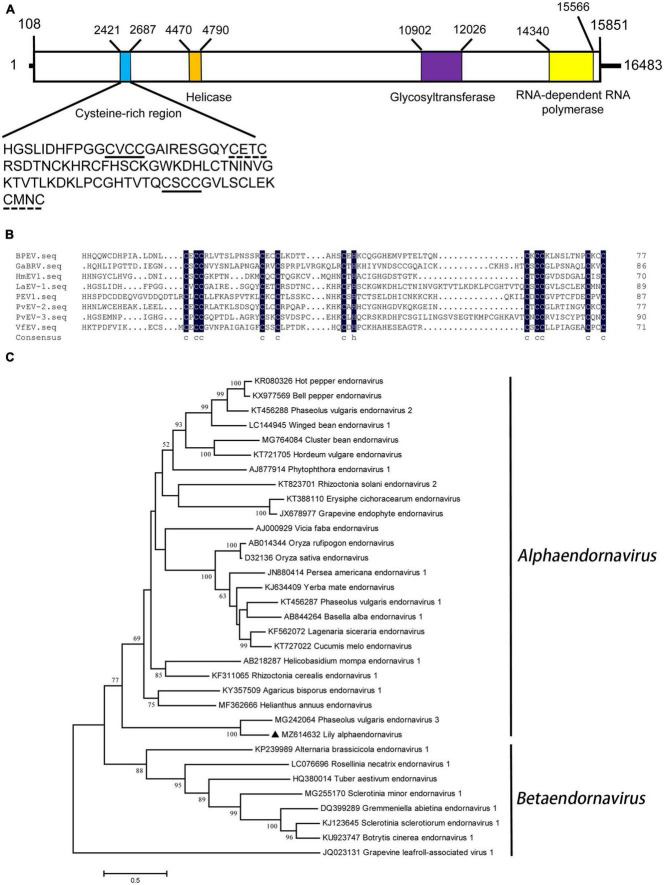
**(A)** Schematic representation of the genomic organization of lily-associated alphaendornavirus 1 (LaEV-1). The nucleotide positions of the protein domain sites were listed. The large box represents the polyprotein encoded by LaEV-1, and the small colored boxes represent the cysteine-rich region (CRR) (blue), whose sequence was presented, helicase domain (orange), glycosyltransferase domain (purple), and RNA-dependent RNA polymerase (RdRp) domain (yellow), respectively. **(B)** Multiple alignment of CRR encoded by LaEV-1 and several other characterized endornaviruses. The sequence of the CRR contains two CxCC signatures marked with solid line, and two signatures marked with dotted line, of which the cysteine at the third character in CxCC signature was substituted. BPEV, bell pepper endornavirus; GaBRV, gremmeniella abietina type B RNA virus; HmEV, helicobasidium mompa endornavirus 1; VfEV, vicia fava endornavirus; PvEV-2, phaseolus vulgaris endornavirus 2; PvEV-3, phaseolus vulgaris endornavirus 3. **(C)** Phylogenetic analysis of the endornaviruses based on the amino acid sequences of RdRp constructed with the maximum-likelihood (ML) method with 1,000 bootstrap replicates. Grapevine leafroll-associated virus 1 was selected as outgroup. Bootstrap values are given by numbers at the relevant nodes in the topology and only bootstrap values of ≥50% were shown. LaEV-1 was indicated with solid black triangle.

The genomic RNA of LaEV-1 was 16,483 nt in length and contained a large open reading frame encoding a polyprotein (5,247 aa, 597.4 kDa) that was preceded and, followed by 5′ and 3′ UTRs of 107 and 632 nt in size, respectively. A BLAST search of the NCBI Conserved Domain Database using the LaEV-1 polyprotein sequence detected conserved domains of a DEAD-like helicase (Hel) superfamily (pfam cl28899, *E*-value = 7.43e^–13^), glycosyltransferase (GT) superfamily (pfamcl10013, *E*-value = 1.02e^–10^) and RdRp superfamily (pfam cl03049, *E*-value = 0.000179842). The nucleotide positions of these three domains in the LaEV-1 genome were determined (Figure 1A). Moreover, a cysteine-rich region (CRR) was identified at nt position 2,421–2,687 by analysis of the LaEV-1-encoded polyprotein (Figure 1A). CRRs have been reported to contain multiple CxCC signatures, with the most conserved signature in all endornaviruses being CxCCG, which could play an enzymatic role in polyprotein processing (Tuomivirta et al., 2009). In the LaEV-1 polyprotein, this CRR region was rich in cysteine residues (19%) and contained two CxCCG signatures. These two CxCC motifs, indicated by a solid line, along with two histidine residues were highly conserved compared with several other recognized species of endornaviruses (Figure 1B), consistent with the results reported by Okada et al. (2011). In addition, two other motifs found in this CRR region, indicated by a dotted line, possibly arose via the substitution of cysteine at the third position in the CxCC signature, which was also found in several other endornaviruses (Okada et al., 2011, 2013).

Sequence comparison with other definite members in *Endornaviridae* showed that LaEV-1 shared the highest nt (44.75%) and aa (35.87%) sequence identities with phaseolus vulgaris endornavirus 3 (PvEV-3) (Table 3), conforming to the species demarcation criteria that members of different species in each genus have an overall nt sequence identity below 75% (Valverde et al., 2019). In the RdRp region, LaEV-1 also shared the highest aa sequence identity (63.81%) with PvEV-3 (Table 3). In addition to the similar genome organization to endornaviruses, an ML phylogenetic analysis of the RdRp protein sequences indicated that LaEV-1 clustered into the alphaendornavirus group closest to PvEV-3 (Figure 1C). In addition, LaEV-1 was not detected in several fungi isolated from symptomatic leaves, suggesting a plant origin of this virus, rather than an epiphytic or endogenous fungal origin (data not shown). Thus, LaEV-1 is likely a member of a novel species in the *Alphaendornavirus* identified in lily.

**TABLE 3:** Nucleotide and amino acid sequence identity values from a comparison of LaEV-1 sequences with the corresponding regions of other endornaviruses.

Virus name	Accession no. (genome/protein)	Genome length (nt)	Sequence identity (%)				
			**Nucleotide**	**Polyprotein**	**Hel**	**GT**	**RdRp**
Vicia faba alphaendornavirus	AJ000929/CAA04392	17,635	39.00	28.71	28.70	–	28.93
Phaseolus vulgaris endornavirus 1	KT456287/ALJ56097	14,072	42.35	27.71	28.05	36.11	30.59
Yerba mate endornavirus	KJ634409/AID67139	13,945	42.43	28.38	No	–	30.33
Cluster bean endornavirus 1	MG764084/AYA60157	12,895	41.82	29.03	38.10	–	32.11
Phytophthora endornavirus 1	AJ877914/CAI47561	13,883	42.55	26.36	22.12	25.00	28.05
Oryza sativa endornavirus	D32136/BAA06862	13,952	39.88	27.33	–	37.14	30.39
Winged bean alphaendornavirus 1	LC144945/BAV69339	14,623	38.49	28.54	28.30	No	30.03
Bell pepper endornavirus	KX977569/AQW41907	14,714	38.47	27.94	26.73	61.54	30.29
Helianthus annuus alphaendornavirus	MF362666/AWK67841	14,663	39.14	27.63	24.76	22.17	29.22
Hot pepper endornavirus	KR080326/ALD49085	14,729	39.76	27.22	27.36	No	29.77
Phaseolus vulgaris endornavirus 2	KT456288/ALJ56098	14,817	39.13	27.49	31.13	No	30.47
Cucumis melo endornavirus	KT727022/ALV83885	15,078	40.09	30.80	No	27.91	34.55
Persea americana endornavirus 1	JN880414/AEX28369	13,459	40.36	29.67	–	–	32.09
Basella alba endornavirus	AB844264/–	14,027	39.34	–	–	–	32.41
Hordeum vulgare endornavirus	KT721705/ALT66307	14,243	39.34	31.07	26.47	–	31.14
Rhizoctonia cerealis endornavirus 1	KF311065/AGY34962	17,486	40.48	31.81	31.73	–	32.47
Oryza rufipogon endornavirus	AB014344/–	13,936	40.69	–	–	–	30.39
Agaricus bisporus endornavirus 1	KY357509/AQM32768	12,730	39.48	28.21	25.71	No	29.59
Lagenaria siceraria endornavirus	KF562072/AHK22715	15,088	39.87	28.68	No	–	32.11
Grapevine endophyte endornavirus	JX678977/AFV91541	12,154	38.70	27.84	32.29	–	30.26
Erysiphe cichoracearum endornavirus	KT388110/ALX17418	11,907	37.76	28.21	No	–	30.21
Helicobasidium mompa endornavirus 1	AB218287/BAE94538	16,614	37.61	28.57	27.10	No	28.46
Phaseolus vulgaris endornavirus 3	MG242064/AXB99512	15,205	44.75	35.87	55.14	–	63.81
Rhizoctonia solani endornavirus 2	KT823701/AMM45288	15,850	39.06	22.69	32.41	–	24.80
Sclerotinia minor endornavirus 1	MG255170/AXX39023	12,626	38.95	23.55	42.86	–	21.65
Botrytis cinerea betaendornavirus 1	KU923747/AOZ66245	11,557	39.16	23.65	22.64	–	23.65
Sclerotinia sclerotiorum betaendornavirus 1	KJ123645/AHN50398	10,513	38.21	23.49	28.85	–	26.98
Alternaria brassicicola endornavirus 1	KP239989/AJA41110	10,290	37.70	22.79	No	–	23.65
Gremmeniella abietina endornavirus 1	DQ399289/ABD73305	10,375	39.00	22.38	22.64	–	22.95
Tuber aestivum endornavirus	HQ380014/ADU64759	9,760	40.65	22.06	–	–	24.35
Rosellinia necatrix endornavirus 1	LC076696/BAT32944	9,639	40.05	24.80	57.14	–	25.25

–, this region has not been characterized; No, no significant similarity.

### 3.3. Characterization of a new polerovirus from lily

**FIGURE 2:**
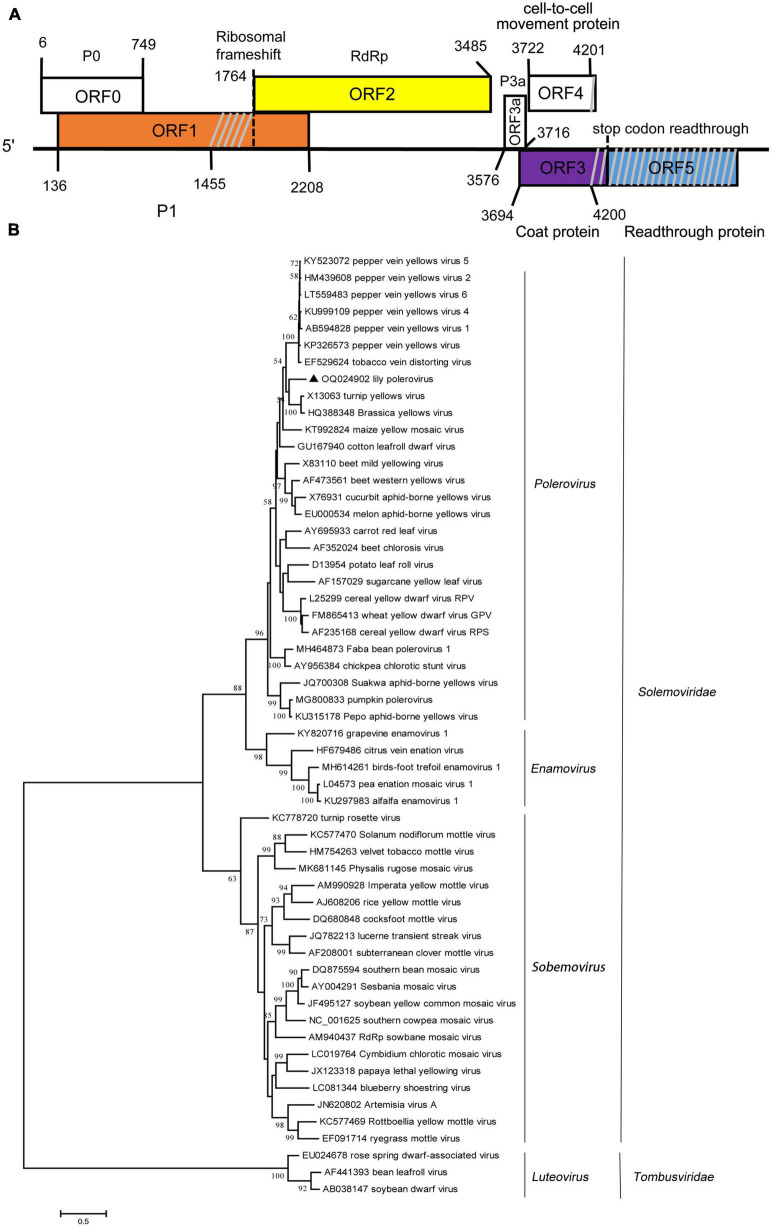
**(A)** Schematic representation of the genomic organization of lily-associated polerovirus 1 (LaPV-1). The nucleotide positions of the protein domain sites were listed. P0, P1 (orange), RdRp (RNA-dependent RNA polymerase, yellow), P3a, cell-to-cell movement protein, coat protein (purple), and reading through protein (blue) were proteins encoded by LaPV-1. Gray slash lines indicate the nucleotide gap in this region. **(B)** Phylogenetic analyses of the viruses in the *Solemoviridae* based on the amino acid sequences of RdRp, constructed with the maximum-likelihood (ML) method with 1,000 bootstrap replicates. Bootstrap values are given by numbers at the relevant nodes in the topology and only bootstrap values of ≥50% were shown. LaPV-1 was indicated with solid black triangle.

The amplified partial genomic RNA sequence of LaPV-1 was 4,021 nt in length, and two definite sequence segments were identified (OQ024902). The genome organization of LaPV-1 was similar to that of poleroviruses in the family *Solemoviridae*, which contain six putative ORFs, and ORF5 failed to be amplified (Figure 2A). The molecular weight of proteins encoding by each ORF was similar to that of proteins encoded by other poleroviruses (Table 4). Moreover, each identified protein shared >10% aa sequence identify with other characterized poleroviruses (Table 4), conforming to the species demarcation criteria for poleroviruses. ORF0 (nt 6–749) translated from the genomic RNA encoded a putative P0 protein. P0 possessed a conserved luteovirus P0 protein superfamily domain (pfam04662 cl04656, *E*-value = 3.89e^–26^). Furthermore, the LaPV-1 P0 protein showed two domains, the amino terminal F box-domain L_58_PLLLX_12_G_75_ and carboxyl terminal conserved sequence K_204_IYGESGX_3_FWR_216_, consistent with most of the studied P0 proteins of poleroviruses (Delfosse et al., 2021). ORF1 (nt 136–2, 208) overlapped with ORF0 and encoded a putative P1 protein, resulting from ribosomal leaky scanning. LaPV-1 P1 exhibited a conserved domain of the peptidase S39 domain–containing protein superfamily (pfam02122 cl09540, *E*-value = 1.60e^–77^). In addition, the E_399_/T_400_ dipeptide site was found in P1, which was considered to be a cleavage site where the polyprotein was cleaved into different functional intermediates (Li et al., 2000). RdRp encoded by ORF2 was produced from ORF1 via a −1 programmed ribosomal frameshift. The synthesis of RdRp might be guided by a heptanucleotide “slip site” (G_1764_GGAAAC_1770_) and a downstream typical *H*-type (2-stem) frameshift-stimulator RNA pseudoknot (nt 1,777–1,804). These two elements are separated by a six-nucleotide linker (Giedroc and Cornish, 2009). RdRp contained a ps-ssRNAv_RdRp-like superfamily (cl40470, *E*-value = 0e + 00), and four conserved catalytic residues, D_375_ and G_469_DD_471_, were identified. A partial ORF3 sequence (nt 3,694–4,200) translated from subgenomic RNA encoded a putative CP protein that also possessed a Luteo_coat superfamily (cl03007, *E*-value = 4.78e^–44^). Furthermore, a nuclear localization signal (P_41_RRRRQSLRRRANR_52_) conserved among CP proteins of poleroviruses was located within the arginine-rich R domain (Haupt et al., 2005). ORF3a (nt 3,576–3,716) encoded the systemic movement protein P3a. ORF3a translation was initiated at a non-AUG codon (ATT), flanked by a favorable translation initiation context with an A at position −3 and a G at +4. In addition, an ORF3a-frame stop codon was found six nucleotides upstream of ATT. The stop codon of ORF3a was located 17 nucleotides downstream of the ORF3 initiation codon, overlapping with ORF3 in the +2 reading frame (Smirnova et al., 2015). A partial ORF4 (nt 3,722–4,201) was embedded within ORF3 in the +1 reading frame and encoded a putative cell-to-cell movement protein. The cell-to-cell movement protein was detected a conserved Luteo_Vpg superfamily (pfam01659 cl03297, *E*-value = 2.048e^–18^), which also existed in two poleroviruses (potato leafroll virus STRAIN1 and barley yellow dwarf virus PAV).

**TABLE 4:** The information on LaPV-1 encoding proteins.

ORF name	Protein name	Number of aa	Mw (kDa)	Amino acid identity (%)
ORF0	P0	246	28.1	27.2–30.7
ORF1	P1	691	–	–
ORF2	RdRp	573	64.1	63.0–72.0
ORF3a	P3a	46	5.0	40.0–67.4
ORF3	CP	167	18.5	60.7–78.9
ORF4	MP	159	17.3	46.5–60.0

Comparison of amino acid sequences of each protein encoded by LaPV-1 with other members in the genus *Poleroviruses* was indicated. –, the information has not been obtained.

The phylogenetic analysis derived from the RdRp aa sequences grouped LaPV-1, brassica yellow virus and turnip yellow virus to one branch separated from 27 other poleroviruses in the *Polerovirus* cluster (Figure 2B). Given these features, LaPV-1 should be considered a divergent new polerovirus in lily.

### 3.4. Genomic characterization of strawberry latent ringspot virus

**FIGURE 3:**
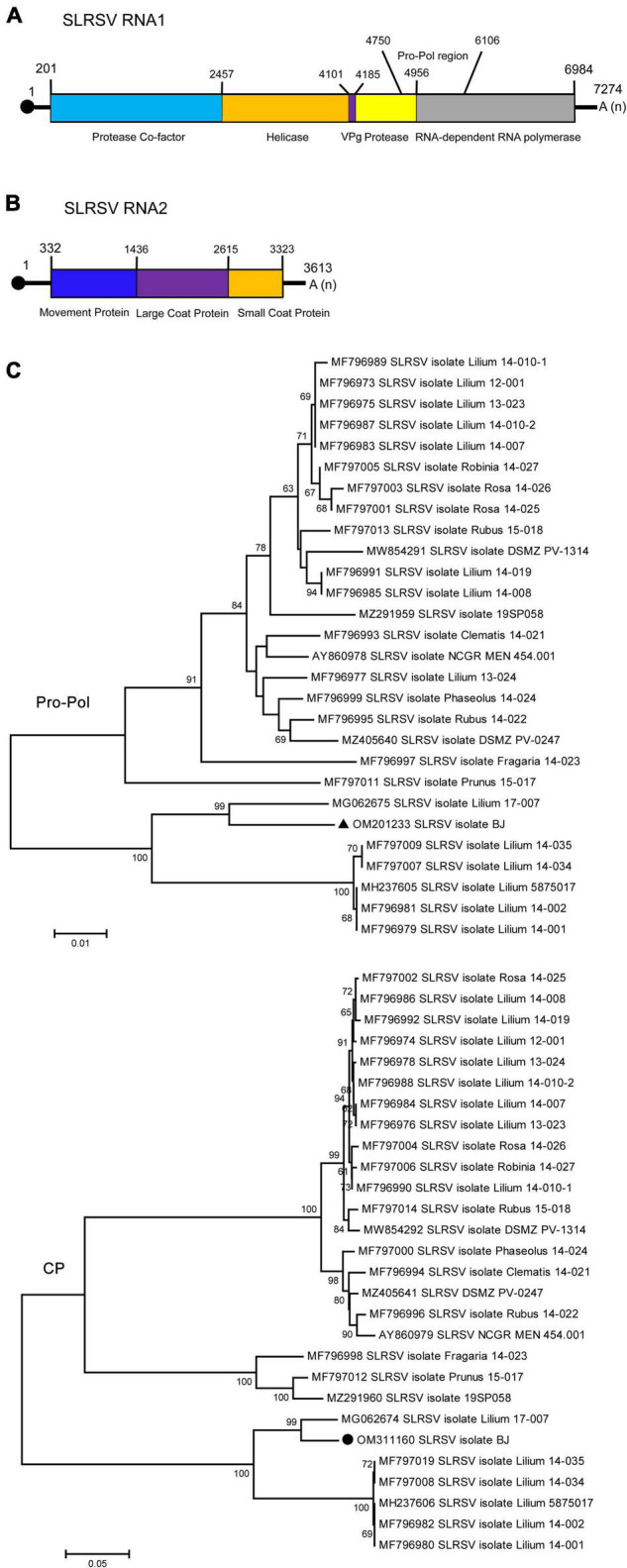
Schematic representation of the genomic organization of strawberry latent ringspot virus (SLRSV). **(A)** The nucleotide positions of the protein domain sites of SLRSV RNA1 were listed. The large box represents the polyprotein encoded by SLRSV RNA1, and the small colored boxes represent the protease co-factor (blue), helicase (orange), VPg (purple), protease (yellow), and RNA-dependent RNA polymerase (gray), respectively. The nucleotide position of Pro-Pol region was also listed. **(B)** The nucleotide positions of the protein domain sites of SLRSV RNA2 were listed. The large box represents the polyprotein encoded by SLRSV RNA2, and the small colored boxes represent the movement protein (blue), large coat protein (purple), and small coat protein (orange), respectively. **(C)** Phylogenetic analyses of the strawberry latent ringspot virus (SLRSV) based on the conserved Pro-Pol region on RNA1 and CP region on RNA2 of all available complete genome sequences constructed with neighbor-joining (NJ) method with 1,000 bootstrap replicates. Bootstrap values are given by numbers at the relevant nodes in the topology and only bootstrap values of ≥50% were shown. RNA1 of SLRSV BJ isolate (OM201233) was indicated with solid black triangle, and RNA2 of SLRSV BJ isolate (OM311160) was indicated with solid black circle.

Strawberry latent ringspot virus, from the genus *Stralarivirus* in the family *Secoviridae*, consists of two positive-sense single-stranded RNA molecules, and is listed as an imported quarantine virus in China. The SLRSV detected in lily in this study is the first report of this virus in China. The RNA1 molecule of the SLRSV BJ isolate was 7,274 nt in length and shared 74.6–79.5% nt sequence identity with other characterized SLRSV isolates at the whole-genome level (Table 5). SLRSV BJ isolate RNA1 (nt 202–6,984) encoded a large polyprotein, which was further cleaved into five proteins by 3C-like proteinases at S/G dipeptide cleavage sites, including protease cofactor (Pro-C), helicase (Hel), virus genome-linked protein (VPg), protease (Pro), and RNA-directed RNA polymerase (Pol) (Figure 3A). The conserved Pro-Pol region at 4,750–6,106 nt position in RNA1 was 452 aa in length, delineated by the “CG” motif of the Pro and the “GDD” motif of the Pol (Figure 3A). A BLASTP search showed that this conserved Pro-Pol region shared 88.1–96.2% sequence identity with Pro-Pol regions encoded by other reported SLRSV isolates, conforming to the species demarcation criteria for *Secoviridae* described in the 10^th^ Report of the International Committee of Viruses (ICTV), in which the conserved Pro-Pol regions of different species had aa sequence identities of more than 80%. The RNA2 molecule of the SLRSV BJ isolate was 3,613 nt in length and shared 75.7–80.9% nt sequence identity with other characterized SLRSV isolates at the whole-genome level (Table 5). The RNA2 molecule also encoded a large polyprotein, which was further cleaved by 3C-like proteinases at dipeptide S/G cleavage sites into movement protein (MP), large coat protein (CP), and small CP (Figure 3B). A BLASTP search showed that the CP shared 64.3–94.3% sequence identity with CP regions encoded by other reported SLRSV isolates, conforming to the species demarcation criteria for *Secoviridae*, in which the CP region of different species had aa sequence identities of more than 75%.

**TABLE 5:** The information on SLSRV-BJ and comparison of SLRSV-BJ sequence with the corresponding regions of other characterized SLRSV genomes.

Genome or protein	Number of nt or aa/Mt (kDa)	Identity (nt, %)	Identity (aa, %)
RNA1	7,274 nt	74.6–79.5	–
5′ UTR	201 nt	5.0–80.0	–
RNA1 encoded polyprotein	2,260/253.9	74.6–84.9	77.3–89.3
Pro-C	752/84.6	60.0–74.0	57.0–76.0
Hel	548/61.2	72.0–81.0	89.0–96.0
VPg	28/3.1	74.0–85.0	100
Pro	257/28.6	76.0–82.0	92.0–97.0
Pol	675/76.1	73.0–87.0	73.0–95.0
3′ UTR	290 nt	2.0–67.0	–
RNA2	3,613 nt	75.7–80.9	–
5′ UTR	332 nt	5.0–85.0	–
RNA2 encoded polyprotein	996/109.9	<76.3	62.0–95.9
MP	368/40.2	21.0–82.0	22.0–99.0
Large CP	393/42.9	63.0–78.0	68.0–95.0
Small CP	235/26.7	58.0–81.0	53.0–92.0
3′ UTR	290 nt	2.0–67.0	–

–, the information has not been characterized.

The above analysis revealed high diversity within the genomes of SLRSV RNA1 and RNA2, with the highest identities of 79.5 and 80.9%, respectively, at the whole-genome nt level, which prompted us to analyze the specific regions where diversity existed. For this purpose, comparisons of the 5′ and 3′ UTR sequences, protein sequences and protein-coding region sequences of the SLRSV BJ isolate with the corresponding sequences of each available full-length characterized SLRSV isolate were performed, and the results showed that diversity existed in almost all regions except for that of the VPg protein encoded by RNA1 (Supplementary Table 2). The nt and aa identities of each region from SLRSV BJ compared with the other 27 characterized SLRSV isolates are shown in Table 5, and the results showed that the Pro-C region presented the lowest similarity at both the nt and aa sequence levels. Detailed analysis revealed that the amino acid sequences in the Pro-C region of other characterized isolates ranged from 700 to 719 aa in length, whereas that of the SLRSV BJ isolate was 752 in length (Supplementary Table 2), suggesting that the difference in sequence length, especially resulting from sequence insertion, could be contribute to the diversity of SLRSV BJ isolate.

Then, the conserved Pro-Pol region of RNA-1 and the CP region of RNA2 were used to determine the relationships among SLRSV isolates according to previous studies involving the phylogenetic analysis of members in the family *Secoviridae* (Thompson et al., 2017; Dullemans et al., 2020). The results showed that both RNA1 and RNA2 of the SLRSV BJ isolate were more closely related to the corresponding sequences of the *Lilium* 17-007 isolate from the Netherlands (MG062675 and MG062674) (Figure 3C). In addition, recombination analysis performed using 28 available SLRSV sequences detected 11 recombinants in SLRSV RNA1 and six recombinants in SLRSV RNA2, and the SLRSV BJ and *Lilium* 17-007 isolates were neither recombinants nor recombinant parents (Supplementary Tables 3, 4).

### 3.5. Characterization of five diversified known lily-infecting viruses

**FIGURE 4:**
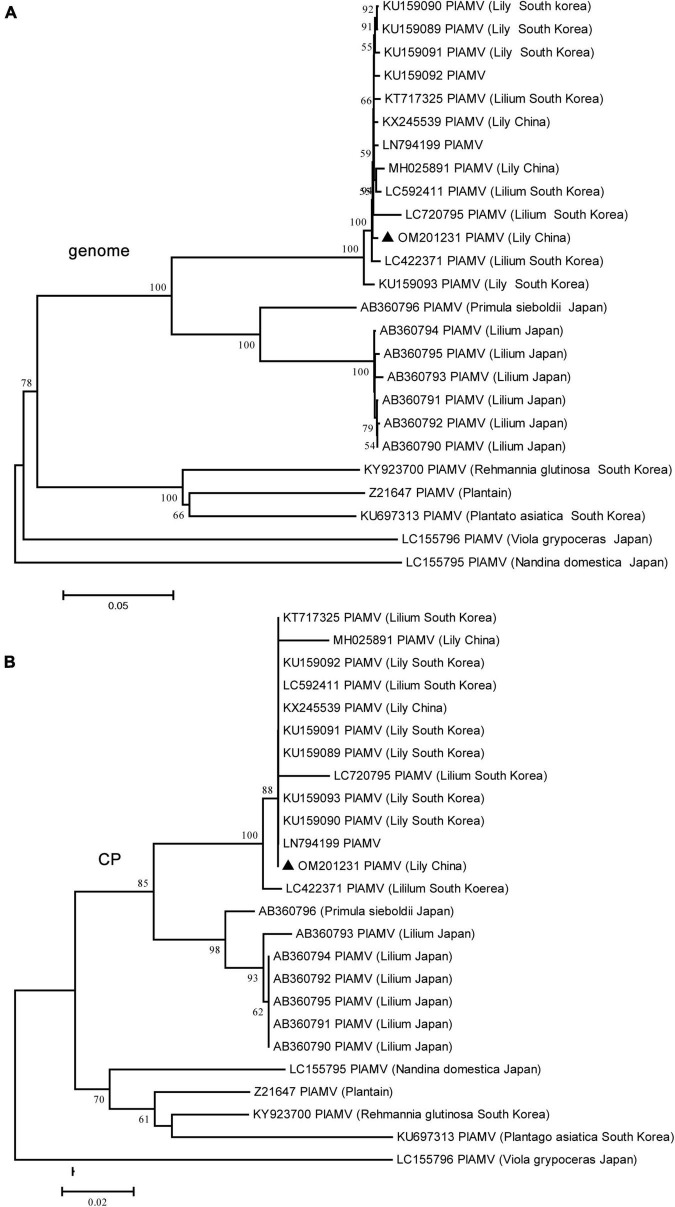
Phylogenetic analyses of plantago asiatica mosaic virus (PlAMV) using the NJ method with 1,000 bootstrap replicates. All available complete genome sequences of PlAMV were collected. **(A)** Based on the complete genome sequences. **(B)** Based on the coat protein (CP) amino acid sequences. Bootstrap values are given by numbers at the relevant nodes in the topology and only bootstrap values of ≥50% were shown. PlAMV BJ strain (OM201231) was indicated with solid blue triangle.

The genome sequence of the LVA BJ isolate was 7,900 nt in length and shared 98.3% sequence identity at the nt level with another characterized isolate (JN127335) from Australia deposited in GenBank. The genome sequence of the LVX BJ isolate was 5,822 nt and shared 98.7% and 98.5% sequence identity at the nt level with two other characterized isolates deposited in GenBank (AJ633822 from the Netherlands and LC335818 from Japan). For the PlAMV BJ isolate, the genome was 6,100 nt in length and shared 77.9–99.6% sequence identity with other available characterized isolates at the nt level. However, no recombination was detected in 25 PlAMV sequences including the newly identified BJ isolate of this study. Interestingly, the phylogenetic tree constructed using these 25 genome sequences or the amino acid sequences of CP encoded by these genomes indicated that these isolates tended to cluster on a host species basis (Figure 4). All isolates from lily clustered into one clade, although four major clades were found in the trees constructed based on the complete genome sequences, and three major clades were found in the trees constructed based on CP protein sequences (Figure 4).

**FIGURE 5:**
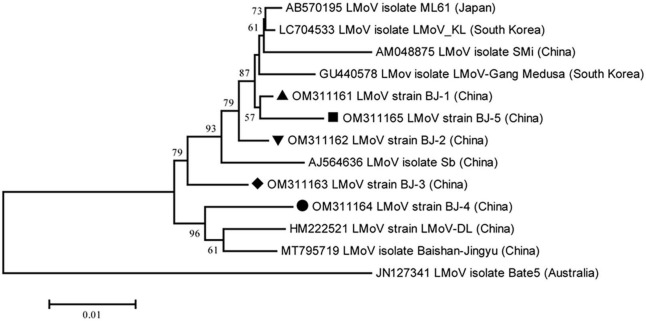
Phylogenetic analysis of the lily mottle virus (LMoV) based on the polyprotein sequences of all available complete genome sequences constructed with NJ method with 1,000 bootstrap replicates. Bootstrap values are given by numbers at the relevant nodes in the topology and only bootstrap values of ≥50% were shown. LMoV BJ-1 (OM311161) was indicated with solid black triangle, LMoV BJ-2 (OM311162) was indicated with inverted solid black triangle, LMoV BJ-3 (OM311163) was indicated with solid black rhombus, LMoV isolate BJ-4 (OM311164) was indicated with solid black circle, and LMoV isolate BJ-5 (OM311165) was indicated with solid black square.

**FIGURE 6:**
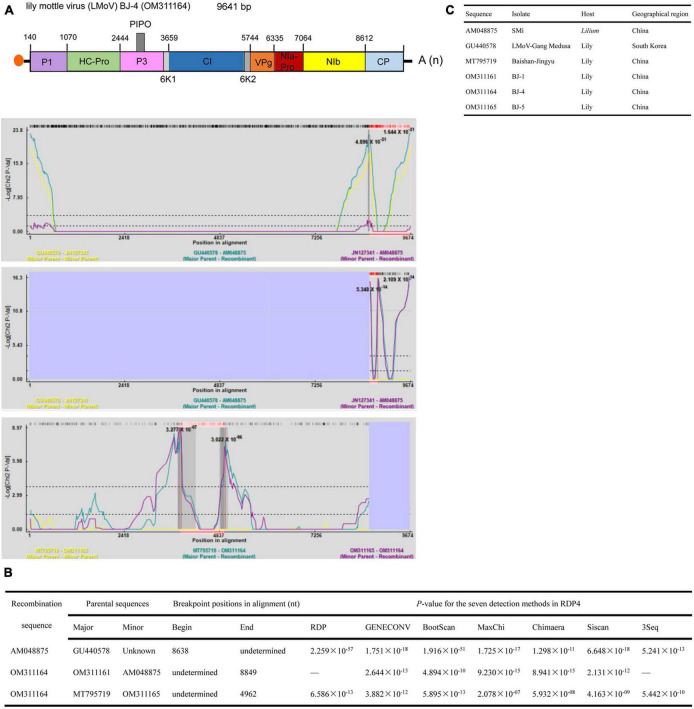
Recombination in the genomic sequences of LMoV analyzed using RDP4. **(A)** Schematic representation of the predicted recombinant LMoV BJ-4 and the MaxChi plots of the recombinants detected by the RDP4 software. **(B)** The relevant information of three predicted recombination events of LMoV genomes. **(C)** The hosts and geographical regions of major and minor parents of the three LMoV recombinants. Major parent, parent contributing larger fraction of sequence; minor parent, parent contributing smaller fraction of sequence; unknown, only one parent and a recombinant need be in the alignment for a recombination event to be detectable. The sequence listed as unknown was used to infer the existence of a missing parental sequence. —, no significant. *P*-value was recorded for this recombination event using this method.

The lengths of the five identified LMoV genomes are listed in Table 2. The pairwise nt sequence identify of each genome and the nt identity of each genome with other characterized isolates are shown in Table 6. The phylogenetic tree constructed using the amino acid sequences of the polyproteins encoded by LMoV isolates revealed that the BJ-1 isolate, BJ-2 isolate, BJ-3 isolate, and BJ-5 isolate were clustered into one group together with other isolates from China, Japan, and South Korea; the BJ-4 isolate was clustered into another group with both the LMoV-DL (HM222521) and Baishan-Jingyu (MT795719) isolates from China; and the Bate5 isolate from Australia was individually clustered into a separate group (Figure 5), suggesting that geographical location may play an important role in the evolution of LMoV from lily plants. Moreover, three recombination events involving two recombinants were identified in all available LMoV isolates. Two recombination events were found in the BJ-4 genome (OM311164) (Figures 6A, B). For one recombination event of BJ-4, the BJ-1 isolate and SMi isolate (AM048875 from China) were predicted to be major and minor parents, respectively, and for another, the Baishan-Jingyu isolate (MT795719 from China) and BJ-5 isolate were predicted to be major and minor parents, respectively. The SMi isolate (AM048875 from China), a minor parent of recombinant BJ-4, was also predicted to be a recombinant, and the LMoV-Gang Medusa isolate (GU440578 from South Korea) was predicted to be its major parent (Figures 6B, C). These results suggested that recombination occurred widely in LMoV, mainly at CI and CP protein regions (Figures 6A, B).

**TABLE 6:** Pairwise nucleotide sequence identify of genomic RNA of each LMoV isolate and the identity of each LMoV isolate with all other characterized isolates.

	Nucleotide identify (%)				
**LMoV isolate**	**BJ-1**	**BJ-2**	**BJ-3**	**BJ-4**	**BJ-5**
BJ-2	99.3				
BJ-3	98.3	98.0			
BJ-4	97.3	97.2	97.3		
BJ-5	99.4	98.9	97.9	97.1	
Other characterized isolates	83.6–99.5	83.6–99.1	83.6–98.0	83.1–97.7	83.4–99.2

**FIGURE 7:**
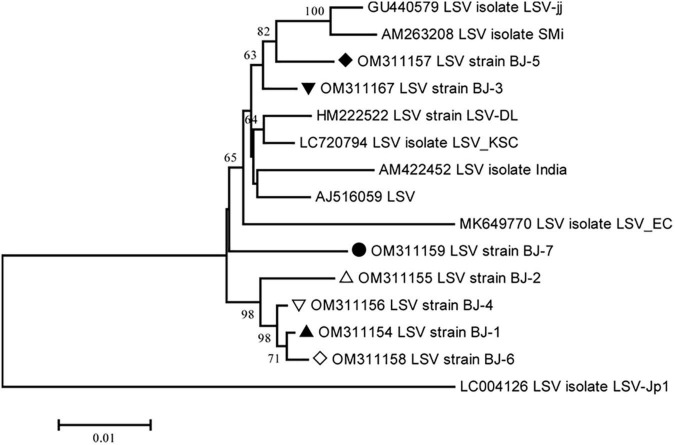
Phylogenetic analysis of the lily symptomless virus (LSV) based on the replicase (refer to RdRp) amino acid sequences of all available complete genome sequences constructed with NJ method with 1,000 bootstrap replicates. Bootstrap values are given by numbers at the relevant nodes in the topology and only bootstrap values of ≥50% were shown. LSV BJ-1 (OM311154) was indicated with solid black triangle, LSV BJ-2 (OM311155) was indicated with triangle, LSV BJ-3 (OM311167) was indicated with inverted solid black triangle, LSV BJ-4 (OM311156) was indicated with inverted triangle, LSV BJ-5 (OM311157) was indicated with solid black rhombus, LSV BJ-6 (OM311158) was indicated with rhombus, and LSV BJ-7 (OM311159) was indicated with solid black circle.

**FIGURE 8:**
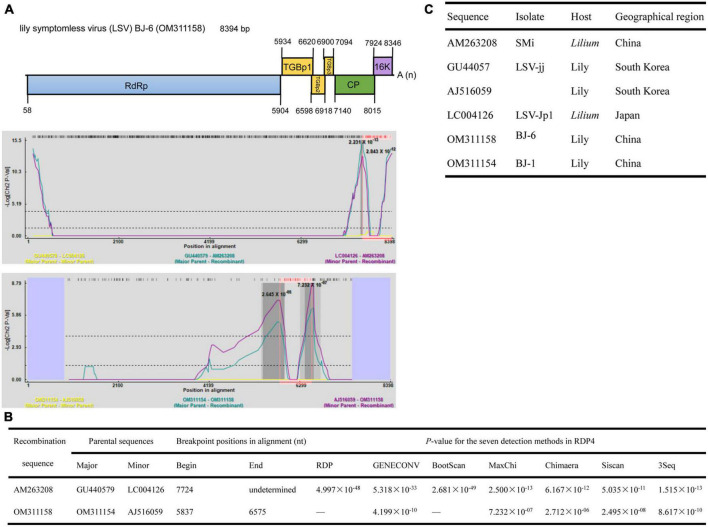
Recombination in the genomic sequences of LSV analyzed using RDP4. **(A)** Schematic representation of the predicted recombinant LSV BJ-6 and the MaxChi plots of the two recombinants detected by the RDP4 software. **(B)** The relevant information of two predicted recombination events of LSV genomes. **(C)** The hosts and geographical regions of major and minor parents of the three LMoV recombinants. Major parent, parent contributing larger fraction of sequence; minor parent, parent contributing smaller fraction of sequence; unknown, only one parent and a recombinant need be in the alignment for a recombination event to be detectable. The sequence listed as unknown was used to infer the existence of a missing parental sequence. —, no significant. *P*-value was recorded for this recombination event using this method.

The lengths of seven identified LSV genomes are listed in Table 2. The pairwise nt sequence identify of each genome and the nt identity of each genome with other characterized isolates are shown in Table 7. The replicase of *Carlavirus* has been considered a principal determinant in the taxonomy of these viruses (Choi and Ryu, 2003). Then, the phylogenetic tree constructed based on replicase amino acid sequences of LSV isolates showed that the newly identified BJ-1, BJ-2, BJ-4, and BJ-6 isolates individually clustered into one group, the BJ-7 isolate individually clustered into one group, and the BJ-3 and BJ-5 isolates clustered most closely with the other six characterized isolates (Figure 7). In addition, recombination analysis of 15 available LSV genome sequences identified two recombination events involving two recombinants (Figures 8A, B). One of these recombinants was the newly identified BJ-6 isolate (OM311158), in which the recombination event occurred between nts 5837 and 6575 (Figures 8A, B). The BJ-1 isolate (OM311154) and the AJ516059 (from South Korea) isolate were predicted to be possible major and minor parents, respectively. The other recombinant was the SMi isolate (AM263208 from China), for which the LSVjj isolate (GU440579 from South Korea) and LSV-Jp1 isolate (LC004126 from Japan) were predicted to be major and minor parents, respectively (Figures 8B, C). These recombinations mainly presented at coding regions, especially at the C-terminus (Figures 8A, B).

**TABLE 7:** Pairwise nucleotide sequence identity of genomic RNA of each LSV isolate and the identity of each LSV isolate with all other characterized isolates.

	Nucleotide identify (%)						
**LSV isolate**	**BJ-1**	**BJ-2**	**BJ-3**	**BJ-4**	**BJ-5**	**BJ-6**	**BJ-7**
BJ-2	99.0						
BJ-3	97.7	97.6					
BJ-4	99.7	99.2	97.6				
BJ-5	97.8	97.2	98.3	97.7			
BJ-6	99.3	98.9	98.0	99.1	97.5		
BJ-7	98.0	97.4	97.6	98.0	97.7	97.7	
Other characterized isolates	85.2–98.5	85.2–98.2	85.3–98.6	85.2–98.4	85.1–98.6	85.2–98.7	85.1–97.9

## 4. Discussion

In this study, combining sRNA-seq, RT-PCR cloning and Sanger sequencing techniques, 12 complete and six nearly full-length genome sequences, including six known and two novel lily-infecting viruses belonging to six genera, were determined in lily samples with virus-like symptoms, revealing viral diversity and complexity in lily plants. Although several contigs matched viruses in the family *Caulimoviridae* (Table 1), the amplification of sequences similar to these viruses failed, possibly due to a low virus titer or sequence contamination. Therefore, it is necessary to use other types of nucleic acid templates, such as total RNA, double-stranded RNA, polyadenylated RNA, or virion-associated nucleic acids purified from virus-like particles, to identify the possible existence of other underlying viral pathogens (Roossinck et al., 2015).

The viral genome of the members in the genus *Polerovirus* in the family *Solemoviridae* contains a polycistronic, positive-sense RNA of 4–6 kb. Poleroviruses share the same basic genome structure, gene functions and expression strategy, applicable to all members within this genus. In this study, a sequence of 4,201 nucleotides was amplified and analyzed, and which showed a similar genome structure and expression strategy with other poleroviruses (Figure 2A). ORF1 encodes a polyprotein consisting of the motifs characteristic of a membrane-anchoring domain, a serine protease, a VPg, and the C-terminal domain. Approximately 310 nucleotides amplified between a serine protease domain and RdRp failed to be characterized (Figure 2A) because this sequence fragment was highly disordered and showed no sequence similarity to any other known virus, consistent with the results of previous studies. Studies on the polerovirus VPg suggest that VPg is variable and intrinsically disordered, with a high frequency of SNPs and nucleotide variation and a large number of positive and negative selection sites at the end of this protein (LaTourrette et al., 2021). The major CP of poleroviruses is encoded by ORF3 of the sgRNA, but if an in-frame readthrough of the amber stop codon occurs, translation is allowed to continue from ORF5 to produce the fusion protein CP-RTD. The RTD region also failed to be amplified, possibly due to the variability in this region and the few contigs obtained.

Poleroviruses are reported to be phloem-specific viruses responsible for infections that usually cause phloem necrosis, yellowing or reddening, streaking, rolling and thickening of leaves and stunting of plants. Furthermore, poleroviruses in mixed infection with potyviruses have been demonstrated to exit the phloem tissue and be readily transmitted by mechanical means (Barker, 1989; Mayo et al., 2000). Additionally, the viruses in such mixed infections have been reported to have synergistic effects and cause enhanced symptoms (Wintermantel, 2005; Zhou et al., 2017). In this study, LaPV-1 was detected in lily plants with symptoms of yellowing, mosaic and leafroll, and mixed infection with the widely distributed potyvirus LMoV (Table 1). Therefore, the effect of LaPV-1 in individual infections or mixed infections with potyviruses (such as LMoV or LVA) on lily production should be further investigated, and it is necessary to monitor whether LaPV-1 could be transmitted by mechanical means in co-infections with potyviruses.

The other novel virus characterized herein was LaEV-1, belonging to the family *Endornaviridae*. The genome of LaEV-1 was successfully assembled from vsiRNAs using HTS, suggesting that host-plant RNA silencing could recognize infecting endornaviruses, consistent with results obtained in other studies (Sela et al., 2012). Additionally, other reports have demonstrated that endornaviruses may encode RNA silencing suppressors to counteract host antiviral silencing (Fukuhara and Gibbs, 2012; Sela et al., 2012). The physiological traits of plants infected with endornaviruses have been reported to be altered (Khankhum et al., 2016; Khankhum and Valverde, 2018). These results indicate that endornavirus infection may have an important effect on host plants. In this study, LaEV-1 was detected in lily plants with yellowing, mosaic, leafroll and stunt symptoms, and co-infection with PlAMV, LMoV, and CMV (Table 1). Therefore, it is necessary to demonstrate the biological role of LaEV-1 in co-infections with other viruses in the future. In addition, plant endornaviruses have been reported to be distributed throughout all tissues and to be transmitted only vertically via seeds, egg cells, and pollen, differing from other pathogenic viruses (Valverde et al., 2019), which reminds us to pay attention to stock quarantine to prevent LaEV-1.

High levels of genetic diversity are a hallmark of RNA virus populations, which contribute to virus evolution for adaptation to new environments (Bentley and Evans, 2018). In this study, eight identified viruses all belonged to single positive-sense RNA viruses, and six viruses, including SLRSV, PlAMV, LVA, LVX, LMoV, and LSV, all exhibited different degrees of genetic diversity. Among these viruses, SLRSV, firstly identified in China, showed the most nucleotide diversity, presenting the highest identity of 79.5% for RNA1 and 80.9% for RNA2 (Table 2), and SLRSV exhibited genetic diversity in the phylogenetic tree (Figure 3C). The diversity of SLRSV was partly due to recombination and sequence insertion, because recombinants involving SLRSV RNA1 and RNA2 were identified (Supplementary Tables 3, 4) and a sequence insertion was newly identified in the Pro-C region of SLRSV BJ RNA1 (Supplementary Table 2). In addition, the result of sequence insertion suggests that RdRp could play an important role in the diversity of SLRSV, which might be attributed to the error-prone nature of its function during viral genome replication (Domingo and Holland, 1997). For PlAMV, isolates from different hosts clustered differentially (Figure 4), indicating that the host may play a role in the diversity of this virus, which was supported by the finding that PlAMV isolates from two host plants showed differences in infectivity and pathogenicity in ornamental lilies (Tanaka et al., 2019). However, for LMoV and LSV, although the phylogenetic analyses also showed the diversity of these two viruses, neither virus presented an obvious pattern related to host plants (Figures 5, 7).

Recombination is considered a significant source of plant virus genetic diversity (Simon-Loriere and Holmes, 2011). Recombination events within the full-length genome sequences of LMoV and LSV were also identified, and one newly identified LMoV isolate and one newly identified LSV isolate were recognized as recombinants (Figures 6, 8), suggesting that recombination could be evolutionarily important in shaping the genomes of LMoV and LSV, consistent with the conclusions drawn from studies of other potyviruses or carlaviruses (Zanardo et al., 2014; Ágoston et al., 2020). For LSV, two recombinants detected were hybrids of parents from different countries (Figure 6), and for LMoV, although the recombinant BJ-4 detected was a hybrid of parents from China, one minor parent of which was also identified as a recombinant, and its parents from other countries. This likely resulted from the frequent lily transportation. Transportation and insect transmission may accelerate recombination to enable recombinants to adapt to new environments.

A previous study showed that recombination in LSV occurred in the region of nt 5,597–7,796, including the C-terminus of the RdRp gene, TGB and almost all CP regions (Singh et al., 2008). However, in this study, two recombinants detected in LSV occurred within the regions of nt 5,837–6,575 and 7,724-undetermined, located in the C-terminus of the RdRp gene, TGBp1, and CP (Figures 8A, B). These differing results possibly resulted from the different numbers of sequences used for analyses. Fortunately, the results of both studies provide evidence that recombination in LSV occurred in the regions involved in virus movement and pathogenicity. For LMoV, recombination at the CP cistron, common in other potyviruses, was also detected (Figures 6A, B), and the CP of potyviruses is reported to be a multifunctional protein and plays important roles in virus life cycle and virus-vector interactions. Thus, the potential risks caused by LSV and LMoV should be monitored, as a mixed infection of different strains could change the virus virulence through recombination.

It was difficult to estimate the damage caused by a single virus identified in this study because of mixed infections (Table 1). Nevertheless, it is necessary to monitor and study the identified lily-infecting viruses, especially novel viruses that may become important potential pathogens, and this opinion has been supported by other reports (Liu et al., 2021). Rapid and accurate detection techniques are considered a prerequisite for viral disease control. The genetic diversity generated in this study prompted us to develop more accurate detection techniques, such as the combination of several HTS methods to obtain more comprehensive viral information and the development of multiplex RT-PCR, multiplex TaqMan or SYBR real-time PCR and immunochromatographic strip test method based on sequences of all available lily-infecting viruses other than sequences of the four common viruses LMoV, CMV, LSV, and PlAMV (Niimi et al., 2003; Zhang et al., 2015; Xu et al., 2022).

## 5. Conclusion

In conclusion, our data suggest that viral genetic diversity widely exists in lily plants, which would provide important information for estimating the potential risk of lily viral disease, for developing more accurate detection techniques, and for cultivation management to prevent the occurrence of viral disease in lily.

## Data availability statement

The datasets presented in this study can be found in online repositories. The names of the repository/repositories and accession number(s) can be found in the article/Supplementary material.

## Author contributions

LC and YL conceived and designed the experiments and wrote the manuscript. LC performed most of the experiments. CG analyzed the data. CY and RS completed amplification of partial sequences. All authors complemented the writing.

## Footnotes

1
https://www.ncbi.nlm.nih.gov/orffinder/

2
www.ncbi.nlm.nih.gov/Structure/cdd/wrpsb.cgi/
