# Supplementary material for: Genetic diversity and phylogenetic characteristics of viruses in lily plants in Beijing
Source: Front Microbiol. 2023 Apr 17;14:1127235. doi: 10.3389/fmicb.2023.1127235 (PMC10149822; doi:10.3389/fmicb.2023.1127235)
Supplement: Supplementary file 1 [file Data_Sheet_1.docx]

Supplementary Material

**Genetic diversity and phylogenetic characteristics in lily plants in Beijing**

**Ling Chen^1^, Cheng Guo^1^, Chenge Yan^2^, Rui Sun^2^ and Yongqiang Li^2*^**

*** Correspondence: Yongqiang Li**: [lyq@bua.edu.cn](mailto:lyq@bua.edu.cn)

# Supplementary Tables

**Supplementary Table 1.** The primer sets used in this study.

| Virus | Reference genome | Product length/bp | Location/nt | Primer | Sequence (5′- 3′) |
| --- | --- | --- | --- | --- | --- |
| LaEV-1 | MZ614632 | 6407 | 417~436 | 1F | GCCGCTTCAATAAGCATGGG |
|  |  |  | 6823~6798 | 1R | GGTTGTATGCCTAGCGTCTTAGCGTC |
|  |  | 5173 | 6704~6726 | 2F | GCTGGTGCCTCTAATAGATCAGG |
|  |  |  | 11876~11850 | 2R | CGGTACCGCTCCGCATCTCAGTATTTC |
|  |  | 2801 | 11656~11682 | 3F | CCCTGATTAGTAGCTTAATAGCTTGGG |
|  |  |  | 14456~14435 | 3R | GCTGGCATACGCCTCAGTATGG |
|  |  | 837 | 837~811 | 5′-RACE | **GATTACGCCAAGCTT**-CCAGATCTTTCTTACTGCAACCAGGCG |
|  |  | 2357 | 14127~14148 | 3′-RACE | **GATTACGCCAAGCTT**-GACATGGATGGGGCTAACGTAG |
|  |  | 300 | 14157~14176 | F-detection | GGAGGGGAAGGAGCTGACTA |
|  |  |  | 14456~1443 | R-detection | GCTGGCATACGCCTCAGTAT |
| LaPV-1 | OQ024902 | 1559 | 394~415 | 1F | ACCAGCACGTGTTTCAACGAGG |
|  |  |  | 1952~1933 | 1R | GGCGGATTCTTCTTCAACGC |
|  |  | 2297 | 1708~1730 | 2F | GCCCCAAGCTCCTTTCGACATTC |
|  |  |  | 4004~3981 | 2R | TAGGCCCGGAGTATTCCAGTAGC |
|  |  | 751 | 751~729 | 5′-RACE | **GATTACGCCAAGCTT**-CCTCAATTGCCTTCTCCGGCCAG |
|  |  | 472 | 3730~3753 | 3′-RACE | **GATTACGCCAAGCTT**-CGGAGACGAGTTCAACGTCGACGC |
|  |  | 553 | 2697~2716 | F-detection | GTAGCCCGGGTTCTGTTTCA |
|  |  |  | 3249~3230 | R-detection | GTTGTCCCGAAACCTCGACT |
| SLRSV RNA1 | OM201233 | 3667 | 820~840 | 1F | GACGCCCTCGCTGATGATTAC |
|  |  |  | 4486~4467 | 1R | ACGAAACAAATGTTGGCGGG |
|  |  | 1113 | 4045~4070 | 2F | CTCTACTCCGCACCCTCTTTGATGAC |
|  |  |  | 5157~5135 | 2R | ATCGTGTAAACGGGTGTCCCGGT |
|  |  | 1616 | 4984~5012 | 3F | GATGCTGAGACCGTTTTCCCAGAAGGTCT |
|  |  |  | 6599~6570 | 3R | GCTGCATCTAAAACCACTACCAGCTCAGGC |
|  |  | 1215 | 1215~1188 | 5′-RACE | **GATTACGCCAAGCTT**-GGGGCTCTGAGAGGAAGGTTCTCCCACC |
|  |  | 998 | 6277~6302 | 3′-RACE | **GATTACGCCAAGCTT**-GCACGCGGGGATAGGTACTTGGCACC |
| SLRSV RNA2 | OM311160 | 2622 | 649~674 | 1F | CTACAGTGGTGCATACGCCAAAGAAG |
|  |  |  | 3270~3246 | 1R | CAGGAATCAGTAACGGAAACCCACC |
|  |  | 1021 | 1021~998 | 5′-RACE | **GATTACGCCAAGCTT**-GACATTGCAGGAGAGCCACCACCC |
|  |  | 543 | 3071~3094 | 3′-RACE | **GATTACGCCAAGCTT**-CACGACGCCACCTGGTCGTTGGAC |
|  |  | 484 | 1604~1623 | F detection | ACTCCGGAGCTCGTTGAAAG |
|  |  |  | 2087~2068 | R detection | ACCCTTAGCAGTCGTTGTCG |
| PlAMV | OM201231 | 2520 | 1795~1816 | 1F | GTGGACTACGAGGGGTTTGGAG |
|  |  |  | 4314~4291 | 1R | TGCCCATAACGGACACGAAAGAAC |
|  |  | 1529 | 3929~3952 | 2F | CTTCAGCCAGTCAAGCCTTAGCTC |
|  |  |  | 5457~5434 | 2R | CACTTCACCCCCAAATCTACACGC |
|  |  | 1964 | 1964~1939 | 5′-RACE | **GATTACGCCAAGCTT**-CCTCTCACCACACCA AGCACACCTCC |
|  |  | 876 | 5225~5249 | 3′-RACE | **GATTACGCCAAGCTT**-ACGTCGCTCCGGAGTGAGGTAGTTG |
|  |  | 713 | 5201~5220 | F-detection | GTAGTGCTCGGTGGTTGTGA |
|  |  | 188 | 5913~5894 | R-detection | GACACAACAACCCTTTCGCC |
| LVA | OM201232 | 2169 | 1345~1371 | 1F | GGAAATCGCGGGCGTCTACTACAATTG |
|  |  |  | 3513~3490 | 1R | CGCCCTTGTGCTTTTGGACAGCTC |
|  |  | 2003 | 2698~2723 | 2F | CCACATCGCGAGGCGATAACATACTG |
|  |  |  | 4700~4674 | 2R | GCTTGAAGTGTACCCGTCCGAGTTGTT |
|  |  | 3249 | 3789~3812 | 3F | CTGGAGTTTCGGAACGTGGAGTAC |
|  |  |  | 7037~7014 | 3R | GAGCTTCTGAATGCGGGGAACCTG |
|  |  | 1808 | 1808~1786 | 5′-RACE | **GATTACGCCAAGCTT**-CTCTTCCAGCAGGGCAGGTTCCC |
|  |  | 1055 | 6846~6870 | 3′-RACE | **GATTACGCCAAGCTT**-CTGGAGGAGCTTGAAGGACCAGAGG |
|  |  | 423 | 4380~4399 | F-detection | GTTGCGTCAAAAGCTCGGTT |
|  |  |  | 4802~4783 | R-detection | GTGGAAGACTCCATGGTGGG |
| LVX | OM311166 | 2967 | 368~394 | 1F | CAAACTCAACGCTCTAGGTCGAAACCC |
|  |  |  | 3334~3309 | 1R | TTGGAGTGGGCATTTCCCCTGAAGCA |
|  |  | 2910 | 2358~2385 | 2F | CATCGCGCTTACTCATACGCAGGTTGTC |
|  |  |  | 5267~5240 | 2R | CAGCTGGAACGCCACATCGAAGAAATTG |
|  |  | 582 | 582~559 | 5′-RACE | **GATTACGCCAAGCTT**-GGGTTTGTACCACCGGGTTGTGGG |
|  |  | 761 | 5063~5085 | 3′-RACE | **GATTACGCCAAGCTT**-GCCCCAGCATCGGGGTTAAGTTG |
|  |  | 327 | 5211~5230 | F-detection | CTGTGGGAAGGGATTGGCAT |
|  |  |  | 5537~5518 | R-detection | AGCAGCAGAGTTGGTGACTC |
| LSV | OM311155 | 4291 | 985~1005 | 1F | GCGCGGAAGAACACACTTTTC |
|  |  |  | 5275~5255 | 1R | AGGCCTCGTAGTCGGATTCAG |
|  |  | 3196 | 4313~4337 | 2F | GAGCTTTTGGCCGCTTCCTATGCAT |
|  |  |  | 7508~7484 | 2R | CCCTGCGATGTCTGACGCTATCTTG |
|  |  | 1188 | 1188~1164 | 5′-RACE | **GATTACGCCAAGCTT**-GATCGTCCGAGTGTTGTCCGTCTCC |
|  |  | 1035 | 7360~7384 | 3′-RACE | **GATTACGCCAAGCTT**-CTGGAACCCACGAGTGCCATGCGAAG |
|  |  | 364 | 7488~7507 | F-detection | ATAGCGTCAGACATCGCAGG |
|  |  |  | 7851~7832 | R-detection | TGATGATCCCCTCGACAGGT |
| LMoV | OM311162 | 2062 | 1159~1180 | 1F | CTTCAACGTCTTCGAGTGTGGG |
|  |  |  | 3220~3198 | 1R | ATGCACGCATTGTGCTAAATCCG |
|  |  | 2504 | 2517~2540 | 2F | GGACACTGATGGAGGATGAGCCCT |
|  |  |  | 5020~4997 | 2R | CTCCGAGTCGCGTAGCTTGTATGG |
|  |  | 5039 | 4339~4363 | 3F | GA CGTTTCAGCAGTTCGTTGCAGTG |
|  |  |  | 9377~9353 | 3R | CTGTATGCCTCTCCGTGTCCTCATC |
|  |  | 1370 | 1370~1346 | 5′-RACE | **GATTACGCCAAGCTT**-CGTTGAGTATGTGTGCCACATGGGC |
|  |  | 573 | 9073~9094 | 3′-RACE | **GATTACGCCAAGCTT**-TGAACACGCAAAACCGACGCTG |
|  |  | 492 | 8880~8899 | F-detection | CCCAGAAGCAGTTTGAGGCT |
|  |  |  | 9371~9352 | R-detection | TATGCCTCTCCGTGTCCTCA |
| CMV | AJ495841 | 895 | 1159~1178 | F-detection | TCTCATGGATGCTTCTCCGC |
|  |  |  | 2053~2034 | R-detection | GCCGTAAGCTGGATGGACAA |

Sequences shown in bold in RACE primers indicate sequences overlapping with the vector and used for In-Fusion cloning. Primers used for virus detection named F-detection and R-detection.

**Supplementary Table 2.** List of the length of the UTRs and proteins of all available full-length SLRSV isolates, and the % of identity in these proteins and their encoded nucleotides compared with those of SLRSV BJ isolate.

| RNA1 Isolate | Accession no. | 5ʹ UTR | % id nt | Poly-protein | % id nt | % id aa | Pro-C | % id nt | % id aa | Hel | % id nt | % id aa | VPg | % id nt | % id aa | Pro | % id nt | % id aa | Pol | % id nt | % id aa | 3ʹ UTR | % id nt |
| --- | --- | --- | --- | --- | --- | --- | --- | --- | --- | --- | --- | --- | --- | --- | --- | --- | --- | --- | --- | --- | --- | --- | --- |
| *Lilium*_5875017 | MH237605 | 205 | 80 | 2219 | 77 | 88 | 711 | 72 | 76 | 548 | 80 | 95 | 28 | 82 | 100 | 257 | 80 | 96 | 675 | 79 | 93 | 375 | 67 |
| *Lilium*_17-007 | MG062675 | 200 | 80 | 2226 | 74 | 90 | 718 | 74 | 77 | 548 | 81 | 96 | 28 | 85 | 100 | 257 | 82 | 97 | 675 | 80 | 95 | 17 | 5 |
| *Rubus*_15-018 | MF797013 | 139 | 57 | 2227 | 71 | 78 | 719 | 62 | 57 | 548 | 75 | 89 | 28 | 76 | 100 | 257 | 76 | 93 | 675 | 74 | 87 | 126 | 30 |
| *Prunus*_15-017 | MF797011 | 231 | 60 | 2228 | 69 | 78 | 719 | 61 | 59 | 548 | 74 | 89 | 28 | 70 | 100 | 257 | 76 | 91 | 676 | 73 | 86 | 357 | 46 |
| *Lilium*_14-035 | MF797009 | 185 | 80 | 2219 | 77 | 88 | 711 | 72 | 76 | 548 | 80 | 95 | 28 | 82 | 100 | 257 | 80 | 96 | 675 | 78 | 93 | 10 | 2 |
| *Lilium*_14-034 | MF797007 | 184 | 79 | 2218 | 77 | 88 | 711 | 72 | 76 | 548 | 80 | 95 | 28 | 82 | 100 | 257 | 80 | 96 | 675 | 78 | 93 | 10 | 2 |
| Robinia_14-027 | MF797005 | 231 | 55 | 2227 | 70 | 79 | 719 | 61 | 59 | 548 | 74 | 89 | 28 | 79 | 100 | 257 | 77 | 92 | 675 | 74 | 87 | 414 | 43 |
| Rosa_14-026 | MF797003 | 234 | 56 | 2227 | 70 | 79 | 719 | 61 | 60 | 548 | 74 | 90 | 28 | 77 | 100 | 257 | 77 | 93 | 675 | 73 | 87 | 66 | 17 |
| Rosa_14-025 | MF797001 | 251 | 53 | 2227 | 70 | 79 | 719 | 61 | 59 | 548 | 73 | 89 | 28 | 79 | 100 | 257 | 76 | 93 | 675 | 74 | 87 | 571 | 39 |
| Phaseolus_14-024 | MF796999 | 242 | 23 | 2227 | 70 | 79 | 719 | 62 | 60 | 548 | 74 | 89 | 28 | 79 | 100 | 257 | 77 | 92 | 675 | 74 | 86 | 309 | 43 |
| Fragaria_14-023 | MF796997 | 215 | 61 | 2227 | 70 | 79 | 719 | 61 | 59 | 548 | 74 | 89 | 28 | 80 | 100 | 257 | 77 | 93 | 675 | 73 | 87 | 376 | 50 |
| *Rubus*_14-022 | MF796995 | 243 | 55 | 2227 | 70 | 79 | 719 | 61 | 60 | 548 | 74 | 89 | 28 | 74 | 100 | 257 | 76 | 92 | 675 | 74 | 86 | 21 | 7 |
| Clematis_14-021 | MF796993 | 56 | 22 | 2227 | 70 | 79 | 719 | 61 | 59 | 548 | 74 | 90 | 28 | 74 | 100 | 257 | 76 | 93 | 675 | 74 | 87 | 100 | 24 |
| *Lilium*_14-019 | MF796991 | 180 | 59 | 2214 | 71 | 79 | 706 | 60 | 62 | 548 | 75 | 89 | 28 | 80 | 100 | 257 | 77 | 92 | 675 | 74 | 87 | 43 | 12 |
| *Lilium*_14-010-1 | MF796989 | 103 | 38 | 2227 | 70 | 79 | 719 | 61 | 60 | 548 | 74 | 89 | 28 | 76 | 100 | 257 | 77 | 92 | 675 | 74 | 88 | 33 | 10 |
| *Lilium*_14-010-2 | MF796987 | 189 | 65 | 2227 | 71 | 80 | 719 | 63 | 60 | 548 | 75 | 90 | 28 | 76 | 100 | 257 | 77 | 93 | 675 | 74 | 88 | 66 | 16 |
| *Lilium*_14-008 | MF796985 | 10 | 5 | 2213 | 71 | 79 | 705 | 64 | 60 | 548 | 75 | 89 | 28 | 79 | 100 | 257 | 77 | 93 | 675 | 74 | 88 | 29 | 9 |
| *Lilium*_14-007 | MF796983 | 157 | 58 | 2227 | 71 | 80 | 719 | 62 | 60 | 548 | 75 | 89 | 28 | 76 | 100 | 257 | 78 | 93 | 675 | 74 | 88 | 35 | 11 |
| *Lilium*_14-002 | MF796981 | 205 | 78 | 2219 | 77 | 88 | 711 | 72 | 76 | 548 | 80 | 95 | 28 | 82 | 100 | 257 | 80 | 96 | 675 | 79 | 93 | 375 | 66 |
| *Lilium*_14-001 | MF796979 | 45 | 18 | 2219 | 77 | 88 | 711 | 72 | 76 | 548 | 80 | 94 | 28 | 82 | 100 | 257 | 80 | 96 | 675 | 79 | 93 | 56 | 17 |
| *Lilium*_13-024 | MF796977 | 221 | 62 | 2208 | 71 | 79 | 700 | 72 | 63 | 548 | 72 | 89 | 28 | 76 | 100 | 257 | 77 | 93 | 675 | 74 | 87 | 40 | 12 |
| *Lilium*_13-023 | MF796975 | 157 | 58 | 2227 | 71 | 80 | 719 | 62 | 60 | 548 | 75 | 89 | 28 | 76 | 100 | 257 | 78 | 93 | 675 | 74 | 88 | 451 | 44 |
| *Lilium*_12-001 | MF796973 | 254 | 57 | 2227 | 71 | 80 | 719 | 63 | 60 | 548 | 75 | 89 | 28 | 76 | 100 | 257 | 78 | 93 | 675 | 74 | 88 | 585 | 37 |
| NCGR MEN 454.001 | AY860978 | 252 | 59 | 2227 | 71 | 79 | 719 | 62 | 58 | 548 | 75 | 89 | 28 | 75 | 100 | 257 | 76 | 92 | 675 | 74 | 87 | 560 | 41 |
| 19SP058 | MZ291959 | 212 | 64 | 2226 | 71 | 79 | 718 | 61 | 59 | 548 | 75 | 89 | 28 | 74 | 100 | 257 | 77 | 93 | 675 | 75 | 86 | 548 | 42 |
| DSMZ PV-0247 | MZ405640 | 239 | 59 | 2227 | 71 | 79 | 719 | 63 | 60 | 548 | 75 | 89 | 28 | 80 | 100 | 257 | 77 | 93 | 675 | 73 | 87 | 556 | 42 |
| DSMZ PV-1314 | MW854291 | 252 | 56 | 2227 | 71 | 79 | 719 | 63 | 60 | 548 | 75 | 90 | 28 | 79 | 100 | 257 | 78 | 93 | 675 | 87 | 73 | 558 | 42 |
| BJ | OM201233 | 201 | 100 | 2260 | 100 | 100 | 752 | 100 | 100 | 548 | 100 | 100 | 28 | 100 | 100 | 257 | 100 | 100 | 675 | 100 | 100 | 290 | 100 |

| RNA2 Isolate | Accession no. | 5ʹ UTR | % id nt | Poly-protein | % id nt | % id aa | MP | % id nt | % id aa | Large CP | % id nt | % id aa | Small CP | % id nt | % id aa | 3ʹ UTR | % id nt |
| --- | --- | --- | --- | --- | --- | --- | --- | --- | --- | --- | --- | --- | --- | --- | --- | --- | --- |
| *Lilium*_5875017 | MH237606 | 378 | 70 | 996 | 77 | 88 | 368 | 79 | 92 | 393 | 75 | 87 | 235 | 76 | 85 | 515 | 46 |
| *Lilium*_17-007 | MG062674 | 327 | 85 | 996 | 80 | 96 | 368 | 82 | 99 | 393 | 78 | 95 | 235 | 81 | 92 | 226 | 70 |
| *Rubus*_15-018 | MF797014 | 228 | 47 | 989 | 67 | 74 | 366 | 75 | 91 | 388 | 64 | 71 | 235 | 58 | 55 | 133 | 32 |
| *Prunus*_15-017 | MF797012 | 373 | 58 | 987 | 68 | 74 | 365 | 77 | 89 | 387 | 64 | 71 | 235 | 60 | 59 | 509 | 39 |
| *Lilium*_14-035 | MF797010 | 20 | 5 | 996 | 77 | 88 | 378 | 80 | 92 | 393 | 75 | 87 | 235 | 77 | 85 | 7 | 2 |
| *Lilium*_14-034 | MF797008 | 20 | 6 | 996 | 77 | 88 | 368 | 80 | 92 | 393 | 75 | 87 | 235 | 77 | 85 | 7 | 2 |
| Robinia_14-027 | MF797006 | 402 | 59 | 989 | 68 | 74 | 365 | 75 | 89 | 389 | 66 | 70 | 235 | 59 | 56 | 219 | 49 |
| Rosa_14-026 | MF797004 | 133 | 28 | 989 | 67 | 73 | 377 | 73 | 86 | 377 | 63 | 68 | 235 | 59 | 56 | 54 | 16 |
| Rosa_14-025 | MF797002 | 416 | 57 | 989 | 66 | 73 | 366 | 74 | 89 | 388 | 63 | 69 | 235 | 59 | 55 | 562 | 40 |
| Phaseolus_14-024 | MF797000 | 293 | 59 | 989 | 68 | 74 | 366 | 76 | 91 | 388 | 67 | 71 | 235 | 59 | 54 | 66 | 20 |
| Fragaria_14-023 | MF796998 | 300 | 60 | 987 | 68 | 74 | 365 | 74 | 89 | 387 | 66 | 69 | 235 | 62 | 59 | 382 | 51 |
| *Rubus*_14-022 | MF796996 | 299 | 59 | 989 | 68 | 74 | 366 | 75 | 91 | 388 | 66 | 72 | 235 | 60 | 54 | 17 | 5 |
| Clematis_14-021 | MF796994 | 22 | 6 | 989 | 67 | 74 | 366 | 75 | 91 | 388 | 66 | 71 | 235 | 59 | 54 | 158 | 38 |
| *Lilium*_14-019 | MF796992 | 266 | 55 | 989 | 67 | 73 | 366 | 74 | 89 | 388 | 65 | 70 | 235 | 60 | 55 | 62 | 18 |
| *Lilium*_14-010-1 | MF796990 | 185 | 42 | 989 | 67 | 74 | 366 | 75 | 89 | 388 | 63 | 70 | 235 | 60 | 56 | 25 | 8 |
| *Lilium*_14-010-2 | MF796988 | 29 | 8 | 989 | 66 | 74 | 366 | 74 | 90 | 388 | 63 | 69 | 235 | 59 | 56 | 24 | 8 |
| *Lilium*_14-008 | MF796986 | 96 | 26 | 989 | 67 | 73 | 366 | 74 | 89 | 388 | 65 | 70 | 235 | 60 | 55 | 28 | 8 |
| *Lilium*_14-007 | MF796984 | 172 | 40 | 989 | 67 | 74 | 366 | 75 | 89 | 388 | 63 | 69 | 235 | 60 | 56 | 72 | 20 |
| *Lilium*_14-002 | MF796982 | 378 | 70 | 996 | 77 | 88 | 368 | 79 | 92 | 393 | 75 | 87 | 235 | 77 | 85 | 413 | 57 |
| *Lilium*_14-001 | MF796980 | 99 | 25 | 724 | 56 | 62 | 96 | 21 | 22 | 393 | 75 | 87 | 235 | 77 | 85 | 56 | 18 |
| *Lilium*_13-024 | MF796978 | 259 | 56 | 989 | 67 | 74 | 366 | 74 | 90 | 388 | 65 | 70 | 235 | 60 | 56 | 442 | 44 |
| *Lilium*_13-023 | MF796976 | 321 | 65 | 989 | 67 | 74 | 366 | 75 | 89 | 388 | 64 | 69 | 235 | 60 | 56 | 442 | 44 |
| *Lilium*_12-001 | MF796974 | 416 | 57 | 989 | 67 | 74 | 366 | 74 | 90 | 388 | 64 | 69 | 235 | 59 | 56 | 609 | 36 |
| NCGR MEN 454.001 | AY860979 | 312 | 61 | 989 | 67 | 74 | 366 | 75 | 90 | 388 | 65 | 71 | 235 | 59 | 53 | 560 | 43 |
| 19SP058 | MZ291960 | 299 | 61 | 988 | 68 | 75 | 366 | 74 | 91 | 387 | 65 | 69 | 235 | 62 | 60 | 548 | 43 |
| DSMZ PV-0247 | MZ405641 | 310 | 60 | 989 | 68 | 74 | 366 | 76 | 90 | 388 | 66 | 71 | 235 | 60 | 54 | 558 | 42 |
| DSMZ PV-1314 | MW854292 | 312 | 63 | 989 | 67 | 74 | 366 | 76 | 91 | 388 | 64 | 69 | 235 | 60 | 55 | 550 | 43 |
| BJ | OM311160 | 332 | 100 | 996 | 100 | 100 | 368 | 100 | 100 | 393 | 100 | 100 | 235 | 100 | 100 | 290 | 100 |

**Supplementary Table 3.** Putative recombination events detected in the SLRSV RNA1 complete genome dataset using RDP4

| Recombination sequence | Parental sequences | | Breakpoint (nt) | | *p*-Value for the seven detection methods in RDP v.4.101 | | | | | | |
| --- | --- | --- | --- | --- | --- | --- | --- | --- | --- | --- | --- |
|  | Major | Minor | Begin | End | RDP | GENECONV | BootScan | MaxChi | Chimaera | Siscan | 3Seq |
| MF797001 | MF797003 | Unknown | undetermined | 1420 | 1.046×10^-59^ | — | 5.472×10^-60^ | 2.567×10^-11^ | 2.271×10^-24^ | 2.050×10^-21^ | 1.513×10^-66^ |
| MF796977 | MF796995 | MF796987 | 133 | 1454 | 3.223×10^-56^ | 7.305×10^-57^ | 2.680×10^-53^ | — | 9.128×10^-15^ | 7.021×10^-36^ | 2.886×10^-13^ |
| MF797001 | Unknown | MF797003 | 2217 | undetermined | 1.577×10^-14^ | 5.175×10^-16^ | 3.735×10^-18^ | 1.849×10^-16^ | — | 5.681×10^-53^ | 9.295×10^-36^ |
| MF797005 | Unknown | MF796983 | 1698 | 6392 | 9.206×10^-16^ | — | 7.072×10^-19^ | 7.683×10^-16^ | — | 1.529×10^-39^ | 1.003×10^-26^ |
| MF796997 | AY860978 | Unknown | 7165 | 7378 | 5.746×10^-15^ | 5.626×10^-11^ | 1.058×10^-07^ | — | — | 2.404×10^-21^ | 1.583×10^-07^ |
| MF796993 | Unknown | MF796995 | 2328 | 5180 | 7.008×10^-17^ | — | 7.037×10^-20^ | 3.438×10^-15^ | 6.923×10^-11^ | 2.441×10^-25^ | 8.792×10^-08^ |
| AY860978 | MF796995 | MW854291 | 6225 | undetermined | 2.163×10^-16^ | 2.428×10^-07^ | 3.339×10^-19^ | — | 6.204×10^-10^ | 1.446×10^-16^ | 2.224×10^-08^ |
| MZ291959 | MW854291 | Unknown | undetermined | 1344 | 3.531×10^-18^ | 9.537×10^-11^ | 3.494×10^-12^ | — | 4.768×10^-10^ | 1.268×10^-22^ | — |
| MF796985 | Unknown | MF797003 | 1059 | 1656 | 8.418×10^-17^ | 1.245×10^-13^ | 6.924×10^-18^ | 6.373×10^-09^ | — | 6.291×10^-13^ | — |
| MF797003 | MF796987 | Unknown | 6436 | 7017 | 8.877×10^-10^ | — | 1.185×10^-08^ | 1.140×10^-07^ | — | — | 5.773×10^-13^ |
| MF796995 | MF796999 | MZ405640 | 2190 | 5194 | — | — | 4.731×10^-07^ | 1.415×10^-08^ | 1.941×10^-08^ | 3.510×10^-18^ | — |

—: not significance

**Supplementary Table 4.** Putative recombination events detected in the SLRSV RNA2 complete genome dataset using RDP4

| Recombination sequence | Parental sequences | | Breakpoint (nt) | | *p*-Value for the seven detection methods in RDP v.4.101 | | | | | | |
| --- | --- | --- | --- | --- | --- | --- | --- | --- | --- | --- | --- |
|  | Major | Minor | Begin | End | RDP | GENECONV | BootScan | MaxChi | Chimaera | Siscan | 3Seq |
| MF796980 | Unknown | MH237606 | 1496 | 3492 | 4.914×10^-41^ | 5.627×10^-36^ | 9.951×10^-32^ | 1.202×10^-28^ | — | 1.090×10^-68^ | 9.664×10^-75^ |
| MF796976 | MF796990 | MF796988 | undetermined | undetermined | 2.324×10^-10^ | — | 7.761×10^-10^ | 1.365×10^-19^ | 3.453×10^-08^ | 9.778×10^-25^ | 4.172×10^-36^ |
| MF797014 | MF797006 | MZ291960 | 3538 | undetermined | 6.011×10^-21^ | 1.555×10^-22^ | 3.916×10^-23^ | — | 3.585×10^-08^ | 8.829×10^-17^ | 5.107×10^-13^ |
| MF796994 | MF797006 | MZ291960 | 3482 | undetermined | 3.637×10^-17^ | 2.373×10^-18^ | 6.132×10^-18^ | — | 1.161×10^-07^ | 1.815×10^-20^ | 9.984×10^-11^ |
| MF797002 | MF797002 | Unknown | undetermined | 1578 | 3.896×10^-10^ | 5.612×10^-08^ | 6.538×10^-10^ | 1.462×10^-13^ | 5.301×10^-15^ | 1.192×10^-16^ | 5.356×10^-18^ |
| MF796986 | MF796992 | MF796974 | undetermined | 1064 | 8.252×10^-14^ | — | 1.292×10^-13^ | 2.505×10^-14^ | 4.603×10^-08^ | — | 1.910×10^-10^ |

—: not significant
